# Elimination of Intraspecific Competition Does Not Improve Maize Leaf Physiological and Biochemical Responses to Topsoil Degradation

**DOI:** 10.3390/plants14162470

**Published:** 2025-08-09

**Authors:** Shan Zhang, Xiaolong Zhang, Zechen Jia, Kaichang Liu, Zhongxiao Guo, Yanjie Lv, Yongjun Wang

**Affiliations:** 1 College of Agronomy, Jilin Agricultural University, Changchun 130118, China; shan502254220@163.com (S.Z.); wssjszsdrmyzy123@163.com (Z.J.); 2 Institute of Agricultural Resources and Environment, Jilin Academy of Agricultural Sciences, Changchun 130033, China; jlguozhx@126.com; 3 State Key Laboratory of Nutrient Use and Management, Shandong Academy of Agricultural Sciences, Jinan 250100, China; feifanxl@163.com (X.Z.); liukc1971@126.com (K.L.)

**Keywords:** topsoil depth, maize planting density, intraspecific competition, nitrogen metabolism enzymes, photosynthesis enzymes, yield variability

## Abstract

Soil degradation limits maize grain yield, but the mechanisms by which leaf functions respond to topsoil depth and their contributions to yield are unclear. We quantified the response mechanisms of leaf functions to topsoil depth with topsoil depths of 10 cm (S_1_), 20 cm (S_2_), 30 cm (S_3_), 40 cm (S_4_), and 50 cm (S_5_) and planting densities of 15,000 plants ha^−1^ (D_1_, the plant spacing was 111.1 cm and there was no mutual influence between individuals) and 75,000 plants ha^−1^ (D_2_). The grain yield in S_1_ was significantly lower than that in S_2_, S_3_, S_4_, and S_5_, and the maximum reductions in yield were 39.7% in D_1_ and 39.1% in D_2_. The coefficients of variation for yield in S_1_ and S_2_ were significantly higher than those in S_3_, S_4_, and S_5_ at both densities and in both years. The net assimilation rate and production efficiency of leaf area, as well as leaf nitrogen and carbon accumulation, all decreased with decreasing topsoil depth. The decreasing topsoil depth significantly reduced the maize leaf net photosynthetic rate, activities of key nitrogen metabolism enzymes, and photosynthesis. Therefore, eliminating intraspecific competition did not reduce the yield loss caused by a reduction in topsoil because leaf nitrogen metabolism and photosynthetic processes were severely limited by the decrease in topsoil depth.

## 1. Introduction

Multiple forms of food insecurity coexist and present a serious global challenge [1]. Owing to both natural and unnatural factors, the incidence of moderate or severe food insecurity in 2020 exceeded that of the previous five years combined, with nearly 12% of the global population (930 million) facing severe food insecurity [2]. Severely degraded agricultural soils are a major cause of food insecurity, with approximately 15.1% of global land suffering from human-induced degradation, and 83.6% of which results from soil erosion [3,4]. Plants adapt to different environmental stresses through various biochemical and physiological mechanisms [5,6]. Therefore, understanding the physiological and biochemical mechanisms of crop adaptation to changes in topsoil depth is important for achieving high and sustainable crop yields.

Based on planting area and production, maize is the largest food crop in China, and it is indispensable in addressing food insecurity [7,8]. However, with the rapid development of intensive agriculture, soil compaction by large agricultural machinery seriously threatens the sustainable production capacity of cultivated soils [9,10,11]. In addition, erosion and loss of topsoil due to improper management practices and climatic environment deterioration have exacerbated soil degradation [4]. Northeast China accounts for 41% of the total maize production in China [8], despite concurrently facing severe soil degradation [12,13]. According to official survey data, the topsoil depth in northeastern China was 50–80 cm in 1950s but only 20–40 cm in 2000 [14]. Reduced topsoil depth damages land resources and reduces soil quality, which can threaten agricultural sustainability [15]. Reductions in topsoil depth significantly reduce corn grain yield and nutrient acquisition capacity [16,17]. The mechanisms underlying limited maize growth and development due to topsoil depth may be multifaceted. Carbon (C) and nitrogen (N) metabolism imbalances are a major consequence of this stress [18], which induces reductions in photosynthesis, canopy area, and C assimilation [19,20,21].

Several studies on roots and soils have elucidated the mechanisms underlying limiting maize production due to reduced topsoil [12,16], but there is currently little evidence from canopy studies. The orderly spatial arrangement of leaves determines canopy structure, greatly influencing solar radiation interception, and thus whole-plant photosynthesis and dry matter accumulation [22]. Optimized canopy structure improves maize grain yield and resource use efficiency [23] by increasing plant density and light interception [24], which increase photosynthetic capacities of fully formed populations [25,26]. Therefore, studies on maize canopy and leaf function can further resolve the mechanisms of maize production limited by topsoil depth.

Increasing planting density can effectively increase maize grain yield [27]. However, it intensifies the competition among individual plants and limits the resources available (such as light, heat, water, and fertilizer) for each plant [28]. By contrast, patterns of individual plants can increase the effective resources available to maize, in turn achieving maximum productivity. The highest grain yields are achieved when maize is grown in its adapted environment and without biotic and abiotic stresses [29]. Therefore, the maize yield response mechanism to topsoil depth can be objectively analyzed by establishing pathways for maize individuals and populations.

We constructed plots with different topsoil depths and maize planting densities in spring-sown maize fields of northeastern China, comparing the responses of maize individuals (15,000 plants ha^−1^) and populations (75,000 plants ha^−1^). We quantified the relation between maize productivity and soil topsoil depth by examining the response mechanisms of maize leaf properties and processes to topsoil depth. The specific objectives were as follows: (1) determine the limiting effects of reduced topsoil depth on maize leaf physiological traits, (2) determine the limiting effects of reduced topsoil depth on maize leaf enzymatic traits, and (3) compare the differences in maize yield responses to leaf physiological and enzymatic traits under individual and population pathways. Therefore, we expect to provide a theoretical basis for sustainable high yields of maize in Northeast China.

## 2. Results

### 2.1. Grain Yield and Yield Components

#### 2.1.1. Grain Yield

**Figure 1:**
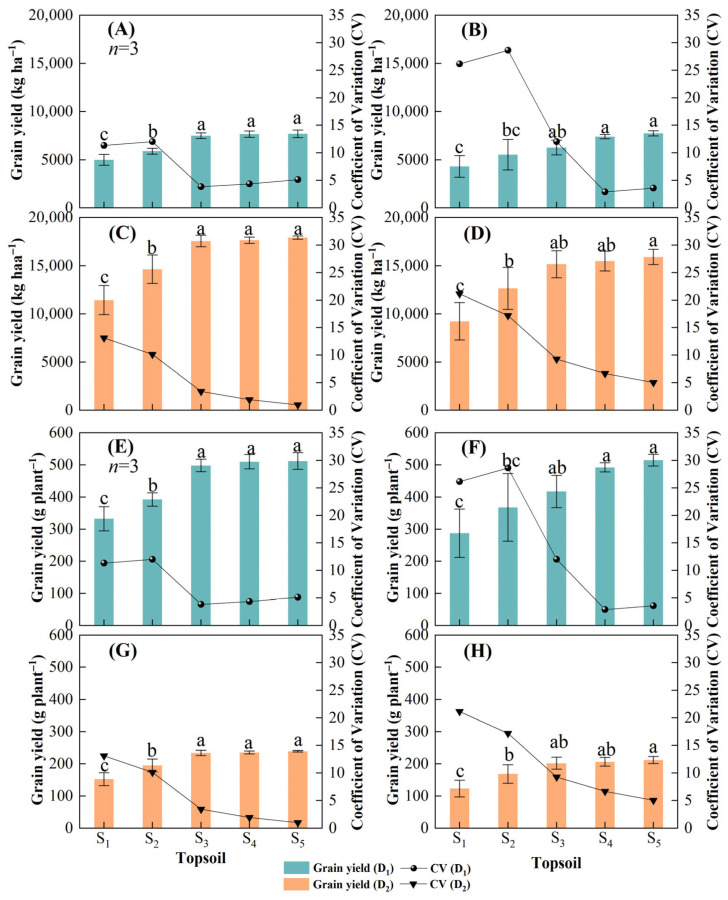
Grain yield and coefficient of variation (CV) at different maize densities and topsoil depths in 2021 and 2022. (**A**–**D**) are the maize yield per unit area and (**E**–**H**) are the maize yield per plant. Grain yield and coefficient of variation at a maize density of 15,000 plants ha^−1^ (D_1_) in (**A**) 2021 and (**B**) 2022 and at a maize density of 75,000 plants ha^−1^ (D_2_) in (**C**) 2021 and (**D**) 2022. Topsoil depth: S_1_, 10 cm; S_2_, 20 cm; S_3_, 30 cm; S_4_, 40 cm; and S_5,_ 50 cm. Values are the mean ± SD. Different lowercase letters above the bars for different treatments indicate significant differences at *p* < 0.05, according to LSD analysis.

Topsoil shallowing impaired maize grain yield in D_1_ and D_2_ (Figure 1). The grain yield in S_1_ was significantly lower than that in S_2_, S_3_, S_4_, and S_5_ in all cases. The maximum reduction in D_1_ yield with reduced topsoil depth was 35.2% in 2021 (Figure 1A) and 44.2% in 2022 (Figure 1B). Similarly, the maximum reduction in D_2_ was 36.2% in 2021 (Figure 1C) and 42.0% in 2022 (Figure 1D). In D_1_, the grain yields in S_3_, S_4_, and S_5_ were significantly greater than those in S_1_ and S_2_ in 2021 (Figure 1A), and the grain yields in S_4_ and S_5_ were significantly greater than that in S_1_ and S_2_ in 2022 (Figure 1B). In D_2_, the grain yields in S_3_, S_4_, and S_5_ were significantly greater than those in S_1_ and S_2_ in 2021 (Figure 1C), and the grain yield in S_5_ was significantly greater than those in S_1_ and S_2_ in 2022 (Figure 1D). In addition, the coefficient of variation (CV) for yield increased significantly with decreasing topsoil depth, and the CVs for S_1_ and S_2_ yields were significantly higher than those for S_3_, S_4_, and S_5_ in all cases. Thus, decreasing topsoil depth not only reduced maize grain yield but also increased uncertainty in grain yield.

#### 2.1.2. Yield Components

**Figure 2:**
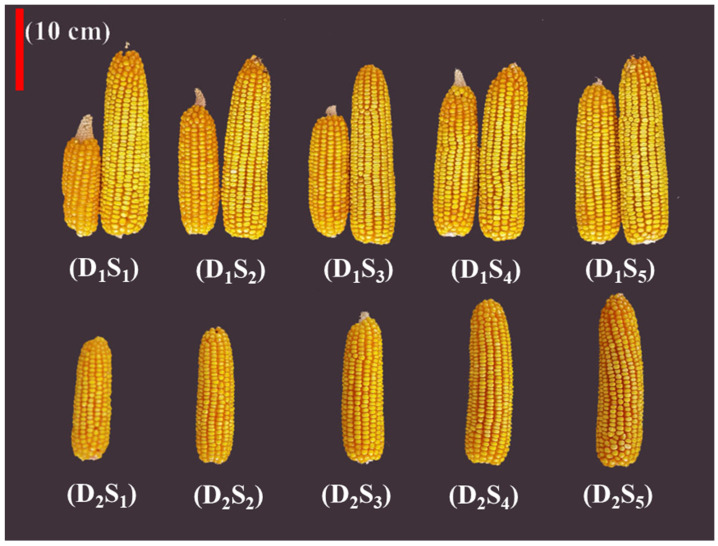
Maize cob characteristics at different maize densities and topsoil depths. Maize density: D_1_, 15,000 plants ha^−1^; D_2_, 75,000 plants ha^−1^. Topsoil depth: S_1_, 10 cm; S_2_, 20 cm; S_3_, 30 cm; S_4_, 40 cm; and S_5_, 50 cm.

The grain yield composition explained the differences in maize yield. With decreasing topsoil depth, the double cob rate of maize decreased significantly in D_1_ and significantly reduced the kernels per plant. The maize per plant in S_3_, S_4_, and S_5_ always maintained 100% double cob rate, whereas the maize per plant in S_1_ and S_2_ had only random appearances of double cobs. In addition, different topsoil depths did not show changes in the larger cob’s traits in double-cob plants but did show significant changes in the smaller cob (Figure 2). With the decrease in topsoil depth, this directly led to differences in the kernels per plant in D1, and the maximum reduction in kernels per plant was 60.7% in 2021 and 80.9% in 2022 (Table 1). In D_2_, no double cobs of maize per plant were observed (Figure 2), and the kernels per plant and 1000-kernel weight were the main factors showing yield differences. In D_2_, the maximum reduction in kernels per plant with decreasing topsoil depth was 33.2% in 2021 and 41.2% in 2022. The maximum reduction in 1000-kernel weight in D_2_ was 21.9% in 2021 and 26.0% in 2022 (Table 1).

### 2.2. Leaf Physiological Parameters

#### 2.2.1. Net Assimilation Rate of Leaf Area

**Figure 3:**
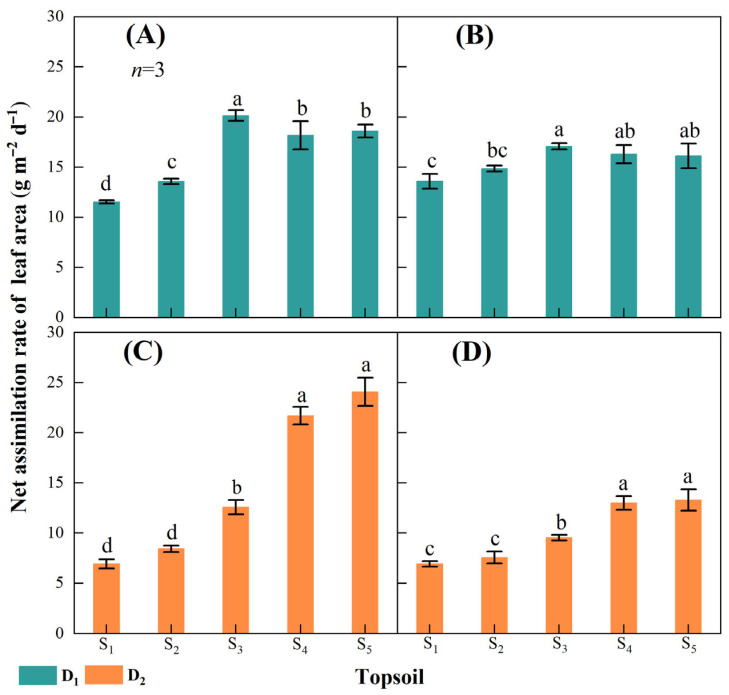
Net assimilation rate of leaf area at different maize densities and topsoil depths in 2021 and 2022. Net assimilation rate of leaf area at the maize density of 15,000 plants ha^−1^ (D_1_) in (**A**) 2021 and (**B**) 2022 and at the maize density of 75,000 plants ha^−1^ (D_2_) in (**C**) 2021 and (**D**) 2022. Topsoil depth: S_1_, 10 cm; S_2_, 20 cm; S_3_, 30 cm; S_4_, 40 cm; and S_5,_ 50 cm. Values are the mean ± SD. Different lowercase letters above the bars for different treatments indicate significant differences at *p* < 0.05, according to LSD analysis.

The two-year study data showed that reducing topsoil depth limited the net assimilation rate (NAR) of leaf area (Figure 3). In D_1_, the maximum decrease in NAR with decreasing topsoil depth was 75.7% in 2021 and 25.8% in 2022 (Figure 3A,B), with the lowest values in S_1_ and the highest in S_3_. In D_2_, the maximum decrease in NAR with decreasing topsoil depth was 247.5% in 2021 and 92.3% in 2022 (Figure 3C,D), with the lowest values in S_1_ and the highest in S_5_.

#### 2.2.2. Leaf Area Production Efficiency

**Figure 4:**
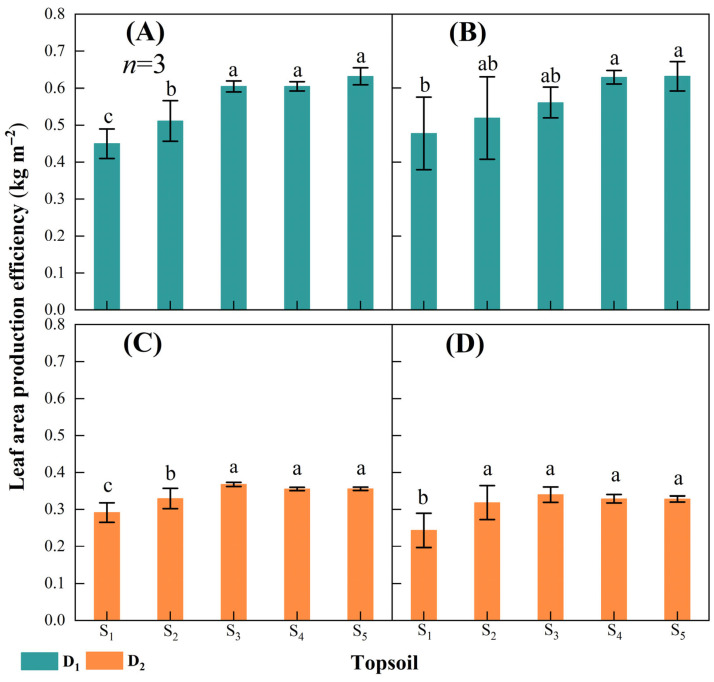
Leaf area production efficiency at different maize densities and topsoil depths in 2021 and 2022. Leaf area production efficiency at a maize density of 15,000 plants ha^−1^ (D_1_) in (**A**) 2021 and (**B**) 2022 and at a maize density of 75,000 plants ha^−1^ (D_2_) in (**C**) 2021 and (**D**) 2022. Topsoil depth: S_1_, 10 cm; S_2_, 20 cm; S_3_, 30 cm; S_4_, 40 cm; and S_5,_ 50 cm. Values are the mean ± SD. Different lowercase letters above the bars for different treatments indicate significant differences at *p* < 0.05, according to LSD analysis.

Topsoil shallowing impaired leaf area production efficiency (LAPE) (Figure 4). In D_1_, the lowest LAPE values were in S_1_ and the highest were in S_5_, and the maximum decrease was 28.8% in 2021 and 24.5% in 2022 (Figure 4A,B). In D_2_, the LAPE levels in S_2_, S_3_, S_4_, and S_5_ were significantly greater than that in S_1_ in both years. The highest values were in S_3_, and the maximum decrease was 18.0% in 2021 and 25.9% in 2022 (Figure 4C,D).

#### 2.2.3. Leaf Nitrogen and Carbon Accumulation

**Figure 5:**
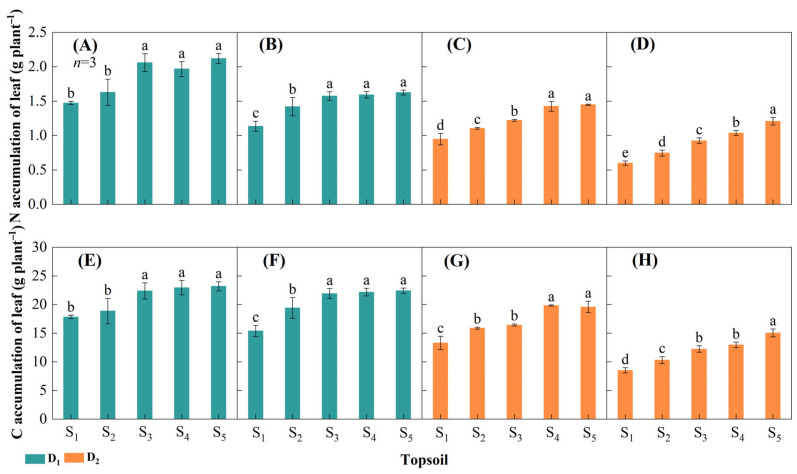
Leaf nitrogen (N) and carbon (C) accumulation at the tassel stage at different densities of maize ands and topsoil depths in 2021 and 2022. Leaf N accumulation at 15,000 plants ha^−1^ (D_1_) in (**A**) 2021 and (**B**) 2022 and at 75,000 plants ha^−1^ (D_2_) in (**C**) 2021 and (**D**) 2022. Leaf C accumulation at D_1_ in (**E**) 2021 and (**F**) 2022 and at D_2_ in (**G**) 2021 and (**H**) 2022. Topsoil depth: S_1_, 10 cm; S_2_, 20 cm; S_3_, 30 cm; S_4_, 40 cm; and S_5,_ 50 cm. Values are the mean ± SD. Different lowercase letters above the bars for different treatments indicate significant differences at *p* < 0.05, according to LSD analysis.

Over the two-year study, decreasing topsoil depth significantly reduced plant leaf N and C accumulation (Figure 5). In D_1_, leaf N accumulation in S_3_, S_4_, and S_5_ was significantly greater than those in S_1_ and S_2_ in both years. The lowest values were in S_1_ and the highest were in S_5_, and the maximum decrease with decreasing topsoil depth was 30.6% in 2021 and 30.1% in 2022 (Figure 5A,B). In D_2_, leaf N accumulation in S_2_, S_3_, S_4_, and S_5_ was significantly greater than that in S_1_. The highest values were in S_5_ in both years, and the maximum reduction with decreasing topsoil depth was 34.5% in 2021 and 50.4% in 2022 (Figure 5C,D). The results for leaf C accumulation are similar to those for N accumulation. In D_1_, the lowest values were in S_1_ and the highest were in S_5_, and the maximum decrease was 23.1% in 2021 and 31.4% in 2022 (Figure 5E,F). In D_2_, the maximum decrease in leaf C accumulation was 32.9% in 2021 and 43.4% in 2022 (Figure 5G,H).

#### 2.2.4. Photosynthesis Rate

**Figure 6:**
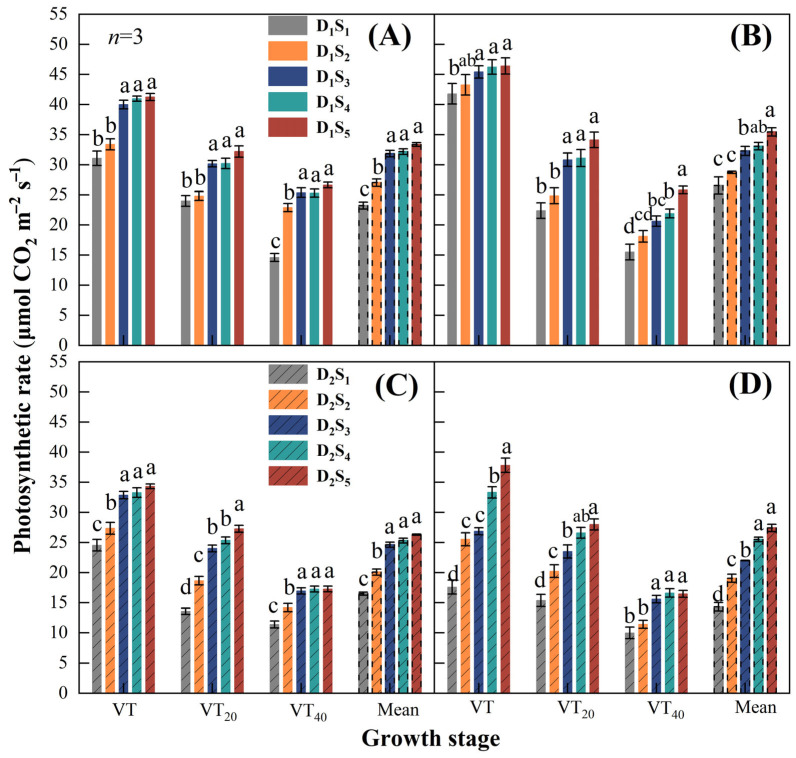
Photosynthesis rate of cob leaf at different maize densities and topsoil depths in 2021 and 2022. Stage-specific and average photosynthesis rate of cob leaf at 15,000 plants ha^−1^ (D_1_) in (**A**) 2021 and (**B**) 2022 and at 75,000 plants ha^−1^ (D_2_) in (**C**) 2021 and (**D**) 2022. VT, VT_20_, and VT_40_ are the tassel stage, 20 d after the tassel stage, and 40 d after the tassel stage, respectively, and the mean is the average of the three stages. Topsoil depth: S_1_, 10 cm; S_2_, 20 cm; S_3_, 30 cm; S_4_, 40 cm; and S_5,_ 50 cm. Values are the mean ± SD. Different lowercase letters above the bars for different treatments indicate significant differences at *p* < 0.05, according to LSD analysis.

The two-year study showed that the photosynthesis rate (Pn) of maize cob leaf gradually decreased with the increase in the growth stage after VT and also with decreasing topsoil depth (Figure 6). In D_1_, the lowest Pn values were in S_1_ and the highest were in S_5_ in both years, and the maximum decreases were 24.7% at VT, 25.5% at VT_20_, and 45.2% at VT_40_ in 2021 (Figure 6A). In 2022, the maximum decreases were 9.9% at VT, 34.5% at VT_20_, and 39.9% at VT_40_ (Figure 6B). In D_2_, the lowest Pn values were in S_1_ and the highest were in S_5_ in both years, and the maximum decreases were 28.5% at VT, 50.3% at VT_20_, and 34.1% at VT_40_ in 2021 (Figure 6C). In 2022, the maximum decreases were 53.5% at VT, 45.1% at VT_20_, and 39.4% at VT_40_ (Figure 6D). The average results of the three monitoring times indicate that the Pn in S_3_, S_4_, and S_5_ was significantly greater than those in S_1_ and S_2_ in both years and at both densities.

### 2.3. Enzyme Parameters

#### 2.3.1. Activities of Key Nitrogen Metabolism Enzymes

**Figure 7:**
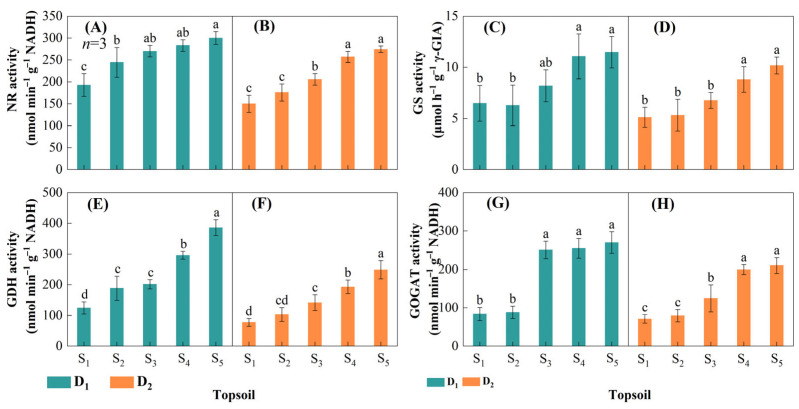
Activities of key enzymes of nitrogen metabolism of cob leaf at different maize densities and topsoil depths in 2021 and 2022. Leaf nitrate reductase (NR) at a maize density of (**A**) 15,000 plants ha^−1^ (D_1_) and (**B**) 75,000 plants ha^−1^ (D_2_); leaf glutamine synthetase (GS) at a maize density of (**C**) D_1_ and (**D**) D_2_; leaf glutamate dehydrogenase (GDH) at a maize density of (**E**) D_1_ and (**F**) D_2_; leaf glutamate synthase (GOGAT) at a maize density of (**G**) D_1_ and (**H**) D_2_. Topsoil depth: S_1_, 10 cm; S_2_, 20 cm; S_3_, 30 cm; S_4_, 40 cm; and S_5,_ 50 cm. Values are the mean ± SD. Different lowercase letters above the bars for different treatments indicate significant differences at *p* < 0.05, according to LSD analysis.

Reductions in topsoil depth significantly reduced NR, GS, GOGAT, and GDH activities in maize leaf at VT. NR, GS, GOGAT, and GDH activities decreased with decreasing topsoil depth, with the lowest values in S_1_ and the highest in S_5_ in all cases (Figure 7). The maximum decrease in NR activity was 35.7% in D_1_ and 45.3% in D_2_ (Figure 7A,B); the maximum decrease in GS activity was 43.6% in D_1_ and 49.8% in D_2_ (Figure 7C,D); the maximum decrease in GDH activity was 67.8% in D_1_ and 69.0% in D_2_ (Figure 7E,F); and the maximum decrease in GOGAT activity was 69.0% in D_1_ and 66.3% in D_2_ (Figure 7G,H).

#### 2.3.2. Activities of Key Nitrogen Metabolism Enzymes

**Figure 8:**
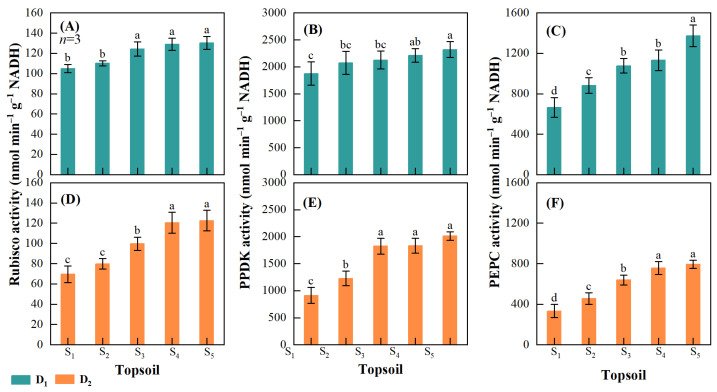
Activities of photosynthetic enzymes of cob leaf at different maize densities and topsoil depths. Leaf ribulose-1,5-bisphosphate carboxylase (Rubisco) activity at a maize density of (**A**) 15,000 plants ha^−1^ (D_1_) and (**D**) 75,000 plants ha^−1^ (D_2_); pyruvate orthophosphate dikinase (PPDK) activity at a maize density of (**B**) D_1_ and (**E**) D_2_; and phosphoenolpyruvate carboxylase (PEPC) at a maize density of (**C**) D_1_ and (**F**) D_2_. Topsoil depth: S_1_, 10 cm; S_2_, 20 cm; S_3_, 30 cm; S_4_, 40 cm; and S_5,_ 50 cm. Values are the mean ± SD. Different lowercase letters above the bars for different treatments indicate significant differences at *p* < 0.05, according to LSD analysis.

Topsoil shallowing impaired Rubisco, PPDK, and PEPC activities in maize leaf at VT. The minimum and maximum Rubisco, PPDK, and PEPC activities in D_1_ and D_2_ were in S_1_ and S_5_, respectively. In D_1_, the maximum decreases in activity were 24.2% for Rubisco, 23.7% for PPDK, and 106.5% for PEPC (Figure 8A–C), while in D_2_, the maximum decreases in activity were 76.3% for Rubisco, 120.3% for PPDK, and 138.2% for PEPC (Figure 8D–F).

### 2.4. Correlations

**Figure 9:**
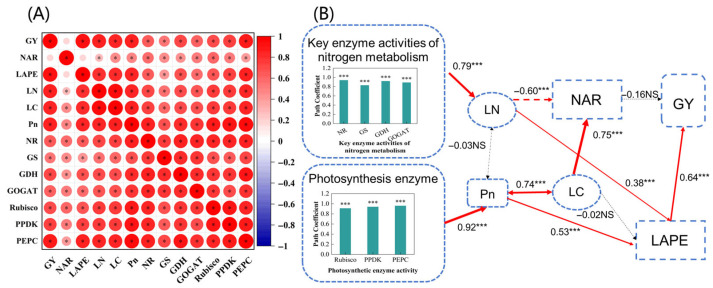
(**A**) Pearson correlation matrix and (**B**) structural equation model of grain yield with main physiological and biochemical indices during the growing stage at different maize densities and topsoil depths in two years. GY, grain yield (g plant^−1^); NAR, leaf area net assimilation rate (g m^−2^ d^−1^); LAPE, leaf area production efficiency (kg m^−2^); LN, leaf nitrogen accumulation (g plant^−1^); LC, leaf carbon accumulation (g plant^−1^); Pn, photosynthetic rate (μmol m^−2^ s^−1^); NR, nitrate reductase (nmol min^−1^ g^−1^); GS, glutamine synthetase (μmol h^−1^ g^−1^); GDH, glutamate dehydrogenase (nmol min^−1^ g^−1^); GOGAT, glutamate synthase (nmol min^−1^ g^−1^); Rubisco, ribulose-1,5-bisphosphate carboxylase (nmol min^−1^ g^−1^); PPDK, pyruvate orthophosphate dikinase (nmol min^−1^ g^−1^); PEPC, phosphoenolpyruvate carboxylase (nmol min^−1^ g^−1^). Circled asterisks in the Pearson correlation matrix in (**A**) indicate significant correlations at *p* < 0.05. Pathways in the structural equation model in (**B**) were significant at *** *p* < 0.001, with NS indicating those that were not significant.

Correlation analysis at different densities and topsoil depths (Figure 9A) showed that grain yield (GY) was not correlated with leaf area NAR but did significantly correlate with LAPE, LN, LC, Pn; key N metabolism enzyme activities (NR, GS, GDH, and GOGAT); and photosynthetic enzyme activities (Rubisco, PPDK, and PEPC).

SEMs (Figure 9B) indicated that key nitrogen metabolism and photosynthesis enzymes, LN, LC, Pn, and LAPE had direct or indirect effects on grain yield (GY) (*p* < 0.001). The LAPE directly influenced yield, whereas other physiological and biochemical indices had an indirect influence on yield. The key N metabolism enzymes directly affected LN, and the photosynthetic enzymes directly affected Pn. The LN and Pn directly affected LAPE and thus GY, and there was a positive interaction between Pn and LC.

## 3. Discussion

### 3.1. Reducing Topsoil Depth Directly Limits Maize Yield

Previous studies found that soil degradation with decreasing topsoil depth negatively affected crop production [13,30]. In this study, maize growth and development were almost unaffected by intraspecific competition at a density of 15,000 plants ha^−1^, and the differences in maize yield were determined by topsoil depth. However, the significant differences in maize yield among topsoil depth treatments were consistent at both 15,000 plants ha^−1^ and 75,000 plants ha^−1^ (Figure 1). Consequently, we conclude that reducing intraspecific competition does not alleviate the limited maize yield imposed by reduced topsoil depth. Therefore, regaining maize yield lost due to decreasing topsoil depth may not be possible by manipulating plant density. This finding is consistent with previous studies. A topsoil depth of 25 cm in eroded soils may be the threshold for sustainable productivity, as crop yields cannot be restored to pre-erosion levels when the erosion depth is greater than 20 cm, although conventional fertilization and irrigation can increase crop yields [31]. In addition, studies have reported the greatest yield decline in the first year of 30 cm topsoil removal for maize, with a 40.9% reduction in grain yield; additionally, a 31.8% reduction in soybean grain yield compared to non-eroded treatment was observed. Continuous manure application combined with crop rotation can restore pre-erosion yield levels, but at a high cost, requiring more than eight years of sustained effort [32]. Therefore, maintaining adequate topsoil thickness can retain high and stable maize yields, while organic treatments and tillage methods incur higher costs and lagging effects. Decreasing topsoil depth also increased the maize yield coefficient of variation (Figure 1), indicating that the yield formation process is highly variable [33]. Although we levelled the ground, ensured seed quality, and performed weed management and fertilizer transport to maintain consistent experimental conditions during the study, maize yields in the shallow topsoil treatment plots were always either randomly very large or very small under the rain-fed conditions. Maize density may have been the primary factor determining double cobs of each maize plant in this study, as there were no double cobs in D_2_. In D_1_, double cobs were typical in S_3_, S_4_, and S_5_; in S_1_ and S_2_, double cobs were more random (Figure 2, Table 1). Our results are in agreement with Li et al., (2021), who reported that rice panicles m^−2^ are mainly responsible for yield reduction following topsoil removal, reporting a panicle m^−2^ reduced by 13.9% compared to CK in rice [34]. Therefore, reduced topsoil depth is associated with the stable formation of crop yields and must be considered in agricultural production. Previous studies have shown that decreased topsoil thickness from 30 to 10 cm resulted in a 9–22% decrease in maize yield [16]. A topsoil depth of 30 cm may be the threshold for normal growth and development of individual maize plants.

However, some studies suggest that the extent of crop productivity reduction with thinning topsoil depends on the crop species. Maize and soybean yield responses to topsoil depth changes were different: maize was more sensitive to topsoil thinning than soybean because of the decreased soil nutrient availability and maize’s inability to extract nutrients from soil [16,35]. This may be due to differences in nutrient and water requirements between soybean and maize, with shallow topsoil limiting maize’s nutrient uptake and utilization while soybean can maintain root growth in shallow topsoil and alleviate nitrogen limitation through biological nitrogen fixation [16]. In this study, the intraspecific competition for nutrients was maximally reduced at 15,000 plants ha^−1^, but significant differences in grain yield for different topsoil depths at 15,000 plants ha^−1^ were consistent with those at 75,000 plants ha^−1^ (Figure 1), indicating that other mechanistic processes may limit maize yield with decreasing topsoil depth.

### 3.2. Reducing Topsoil Depth Limits Leaf Production Capacity

Leaves are the primary organs for accumulating photosynthetic assimilates and rely on solar radiation for yield formation [36,37]. The number and area of maize leaves increased rapidly during the vegetative stage and reached a maximum at the tassel stage. Moreover, adverse stresses during the maize vegetative stage result in greater yield loss than those during the reproductive stage [38]. Compared with other biological traits, leaf growth is prospective and readily apparent, even if it is not the only or the most important factor [39]. The leaf area NAR increased and then decreased in the 10–50 cm topsoil depth range at 15,000 plants ha^−1^, whereas it increased from S_1_ to S_5_ at 75,000 plants ha^−1^ (Figure 3). This might be due to the existence of other dominant pathways for biomass assimilation for LA in the absence of intraspecific competition. In previous studies, root growth was closely related to an increase in LA [40,41], and more biomass may be transformed into the root system with increases in LA. Previous studies have shown that increased response was in fact correlated with increased root growth below 30 cm depth [42]. Thus, ensuring a certain degree of topsoil depth is undeniably important to stabilize the matter assimilation function of maize leaves.

Leaf area is considered to play an essential role in grain yield formation [43,44,45]. In this study, grain accumulation encompassed the process of biomass accumulation after VT, and reduced topsoil depth reduced the maize leaves’ contribution to grain filling. Reduced topsoil depth decreased LAPE, and the differences in LAPE among topsoil depth treatments were greater at 15,000 plants ha^−1^ than at 75,000 plants ha^−1^ (Figure 4). This might be explained by the low degree of mutual shading by maize leaves at 15,000 plants ha^−1^, amplifying the limiting effect of reduced topsoil depth on LAPE. Reduced topsoil depth limits leaf photosynthetic capacity and radiation use efficiency, thereby decreasing LAPE [39,46].

### 3.3. Reducing Topsoil Depth Limits Leaf Nutrient Accumulation and Photosynthetic Rate

Leaf N and C contents are closely related to the assimilation and production of matter, with C and N accumulations being the most important basis for transporting nutrient matter to other organs [47]. Previous studies show that N storage and transport in leaves supports protein content increased in sweet corn kernels [48]. Approximately 50% to 70% of leaf N is remobilized, contributing 22% to 46% of grain N [49,50,51]. Leaves can automatically balance sink–source relationships, and excess accumulated C and N is actively transferred to the rest of the organ [52,53,54]. Previous studies have shown that the percentage of N uptake from topsoil to plant N uptake ranged from 54.2 to 62.4%, and topsoil removal reduced total N uptake by 27.8% [34]. In this study, reducing topsoil depth limited maize leaf N and C accumulation (Figure 5); therefore, limited C and N accumulation may underpin limited leaf assimilation and matter production.

Leaf photosynthetic rate is directly related to leaf N content [55]. Maintaining relatively high photosynthetic leaf activity contributes to increases in maize yield [56]. In this study, reducing the topsoil depth significantly reduced the photosynthetic rate of maize cob leaf during the reproductive stage (Figure 6). This is consistent with previous studies [35], in which topsoil removal substantially reduced aboveground dry matter at later stages of vegetative growth, with plant height at harvest and photosynthetic rates during reproductive stages also reduced. The improved root architecture in deep topsoil is a key physiological basis for achieving high yields in densely planted maize populations [57]. At relatively low soil depths, crop root development is restricted, reducing nutrient uptake and utilization and potentially inhibiting photosynthesis by disrupting the leaf photosystem and reducing activities of key enzymes [58].

### 3.4. Reducing Topsoil Depth Limits Leaf Enzyme Activities

Drought stress significantly reduces key N metabolism enzymes’ activities and thus decreases N accumulation and transport [59]. Moreover, N affects crop metabolic processes and is intricately linked to plant physiological events [60,61]. In addition, the activities of key N metabolism enzymes are closely related to photosynthesis [62]. In this study, topsoil degradation influenced the activities of NR, GS, GDH, and GOGAT, the key N metabolism enzymes in maize leaves (Figure 7). Key leaf N metabolism processes are inhibited when topsoil depth is reduced, which may result in premature leaf senescence and reduced internal N translocation and productivity in maize [18,63].

Photosynthesis is intimately related to the soluble protein content, especially through the Rubisco, PEPC, and PPDK enzymes [64,65,66]. Rubisco is a key enzyme in photosynthetic C assimilation, and PEPC and PPDK have regulatory roles in plant photosynthesis and response to environmental stress [67,68]. Photosynthetic enzymes serve as a N storage reservoir at the early grain filling stage, and their degradation is critical in reducing Pn during the later grain filling stage [69]. In this study, topsoil degradation influenced the activities of maize Rubisco, PEPC, and PPDK (Figure 8), explaining the decrease in Pn.

### 3.5. Physiological Processes and Mechanisms

In this study, there were significant correlations between all physiological and biochemical indices (excluding NAR and yield) (Figure 9A), but there was no significant interaction between LN and Pn (Figure 9B). According to previous studies, there is a trade-off between leaf N reallocation and leaf Pn during post-silking stages for a given N content because as more N is reallocated from leaves to grains, less N remains in the leaves to support photosynthesis [70,71,72]. This may explain why LN can directly increase LAPE. Although increased N reallocation is important to support grain growth, it may concomitantly result in lower leaf N content, in turn accelerating leaf senescence and leading to decreases in leaf N content, green leaf area, and canopy photosynthesis [73,74]. Therefore, LN and Pn may exist in a dynamic state of mutual balance, showing neither stable positive nor negative effects [75]. In this study, photosynthetic enzymes affected LC and NAR by increasing Pn but ultimately did not affect yield (Figure 9B). Consequently, balancing the accumulation and distribution of substances during the vegetative and reproductive growth stages of maize may further increase maize yield under deep soil conditions.

In summary, increasing the activities of key N metabolism and photosynthesis enzymes in maize cob leaf can effectively increase maize yield at different planting densities and topsoil depths. The physiological mechanisms involve Pn, LN, and LC and thus increase the LAPE of maize.

## 4. Materials and Methods

### 4.1. Site Description

**Figure 10:**
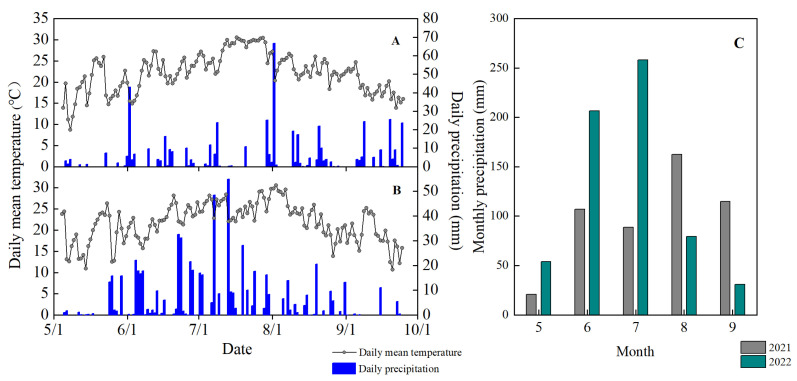
Daily mean temperature and daily precipitation in (**A**) 2021 and (**B**) 2022 and (**C**) monthly precipitation during maize growth in 2021 and 2022 at the study site in Jilin Province, China.

This study was conducted at the Gongzhuling Experimental Station of the Jilin Academy of Agricultural Sciences, Jilin Province, China (43°52′ N, 124°81′ E; 206 m a.s.l.). The region is in temperate and cold temperate zones with humid and semi-humid climates. It is cold and dry in winter and warm and wet summers. The average annual duration of sunlight in the experimental region is 2624 h; the total solar radiation is 5551 MJ m^−2^; the frost-free stage is 144 d; the average annual temperature is 6.7 °C; and the average annual precipitation was 572.7 mm over the past 15 years. This climate combined with good soil quality is highly conducive to maize production. The specific meteorological data of the experimental site during the study period are represented in Figure 10. The average temperatures in 2021 and 2022 were 22.2 °C and 21.8 °C, respectively, and the total precipitation was 513.6 mm and 628.8 mm, respectively (Figure 10A,B), with the precipitation in 2021 being more concentrated in the later stages of maize reproductive life (August–September) (Figure 10C), which supported grain filling. The soil is a typic Mollisols [32], known locally as “black soil”, and had the following chemical properties in the 0–20 cm tillage layer: organic matter, 15.1 g kg^−1^; alkaline N, 192.9 mg kg^−1^; fast-acting phosphorus, 10.1 mg kg^−1^; fast-acting potassium, 292.3 mg kg^−1^; and pH, 7.9.

To create an independent system of test plots (1.8 m × 4.0 m = 7.2 m^2^), 50 cm high iron frames and plastic sheets were used. In addition, a 200-mesh (0.074 mm) nylon net at the bottom of plots was used to simulate limited soil plow pan on maize root growth. Before the experiment, cultivated soil was removed to depths of 10, 20, 30, 40, and 50 cm. Iron frames were inserted vertically, and root-limiting nets were laid horizontally at the different soil depths, with the soil below the net interface compacted. Then, the area above the net was filled with the removed soil, watered, and allowed to settle (Figure S1).

### 4.2. Experimental Design

The experiment had a 2 × 5 (two plant densities and five topsoil depths) factorial design with 10 treatment combinations. Each treatment was replicated in three plots in a complete randomized block design. The maize cultivar was Fumin985. Plant densities were 15,000 plants ha^−1^ (the D_1_plant spacing was 111.1 cm and there was no mutual influence between individuals) and 75,000 plants ha^−1^ (D_2_), and the topsoil depths were 10 cm (S_1_), 20 cm (S_2_), 30 cm (S_3_), 40 cm (S_4_), and 50 cm (S_5_) (Table 2). The typical local planting density was 75,000 plants ha^−1^ and the plant spacing was 22.2 cm; the plant spacing of 15,000 plants ha^−1^ was 111.1 cm, and is considered a pattern with no intraspecific competition. All treatments received the same level of fertilizers (N, 200 kg ha^−1^; P_2_O_5_, 90 kg ha^−1^; K_2_O, 100 kg ha^−1^), with N, P, and K applied once as a base fertilizer before sowing.

### 4.3. Measurements

#### 4.3.1. Grain Yield

At the maturity stage, all maize cobs in the plots with 15,000 plants ha^−1^ were harvested and then dried in the laboratory. For 75,000 plants ha^−1^, ten cobs were collected from the middle row of each plot and then dried in the laboratory. All kernels per cob were considered maize grain yield (g plant^−1^), with the grain moisture content adjusted to 14%. In cases of double cobs per plant at 15,000 plants ha^−1^, both were used in calculating grain yield.

#### 4.3.2. Leaf Area Net Assimilation Rate and Production Efficiency

Leaf area (LA) was determined twice per season at critical crop development stages (tassel stage, VT; maturity stage, R6) using the length and width coefficient method, with LA calculated as follows:

LA (cm^2^) = length (cm) × width (cm) × 0.75 (1)

At the same two crop development stages, the entire aboveground part of the plant was collected, including the entire stem, leaf, and other parts, and oven-dried at 85 °C to a constant weight. The net assimilation rate (NAR) of LA was calculated as follows [76,77]:

NAR = ΔDM/ΔLA/ΔT × 10^−4^ (2)

where NAR is the net assimilation rate of LA (g m^−2^ d^−1^); ΔDM is the difference in dry matter weight of plants between emergence of seedlings and VT (g plant^−1^); ΔLA is the difference in leaf area between emergence of seedlings and VT (cm^2^ plant^−1^); ΔT is the number of days between emergence of seedlings and VT (d). The critical dates are provided in Table S1.

Leaf area production efficiency (LAPE) was calculated as follows:

LAPE = Y_G_ × (1 − 0.14)/LA_M_ × 10^−7^ (3)

where LAPE is the leaf area production efficiency (kg m^−2^); Y_G_ is the grain yield (g plant^−1^); LA_M_ is the mean leaf area between VT and R6 (cm^2^ plant^−1^). The LAPE was calculated using grain weight without water (14%).

The LAs for the VT and R6 stages are shown in Table S2, and the dry matter weights for the V6, VT, and R6 stages are shown in Table S3.

#### 4.3.3. Leaf Nitrogen and Carbon Accumulation

At the VT stage, maize leaf was dried, weighed, and pulverized, and N and C concentrations were measured using an elemental analyzer (Elementary Various MICRO cube, Elementar, Langen Selbold, Germany). Leaf C and N accumulation were calculated as follows:

N accumulation (g plant^−1^) = δ_(N)_ × DW_(Leaf)_ (4)

C accumulation (g plant^−1^) = δ_(C)_ × DW_(Leaf)_ (5)

where δ_(N)_ and δ_(C)_ are N and C concentrations (%), respectively, and DW_(Leaf)_ is the leaf dry matter (g plant^−1^).

#### 4.3.4. Leaf Nitrogen and Carbon Accumulation

The net photosynthetic rate (Pn) of cob leaf was measured by randomly selecting five plants from each plot at the silking stage (R1) and at 20 d (R1_+20_) and 40 d (R1_+40_) after the silking stage. Plants were sampled between 1000 and 1200 using an LI-6400 portable photosynthetic instrument (LI-COR Inc., Lincoln, NE, USA). During the measurement period, the light intensity was kept stable at 1700 ± 50 μmol m^−2^ s^−1^.

#### 4.3.5. Determination of Activities of Key Enzymes of Leaf Nitrogen Metabolism and Photosynthesis

At the VT stage, a fresh portion of cob position leaves was retained and stored at –80 °C to determine the activities of the following key leaf N metabolism enzymes: nitrate reductase (NR), glutamine synthetase (GS), glutamate dehydrogenase (GDH), and glutamate synthase (GOGAT). The activities of the photosynthetic enzymes ribulose-1,5-bisphosphate carboxylase (Rubisco), pyruvate orthophosphate dikinase (PPDK), and phosphoenolpyruvate carboxylase (PEPC) were also measured at the VT stage. All enzyme activities were measured using kits (Suzhou Michy Biomedical Technology Co., Ltd., Suzhou, China) and an enzyme labeler (Spectramax i3x, Molecular Devices, LLC, Salzburg, Austria). Kit instructions briefly describing the assay principle and defining units of activity can be downloaded from the company’s website [78].

Nitrate reductase catalyzes the reduction of nitrate to nitrite: NO_3_^−^ + NADH + H^+^ → NO_2_^−^ + NAD^+^ + H_2_O. NADH has a maximum absorption peak at 340 nm, and NR activity is expressed by determining the rate of NADH reduction. Therefore, one unit of enzyme activity (nmol min^−1^ g^−1^) is defined as the catalytic reduction of 1 nmol of NADH per min per g of fresh weight sample.

Glutamine synthetase catalyzes the synthesis of glutamine from ammonium and glutamic acid in the presence of ATP and Mg^2+^; glutamine is further converted to γ-glutamyl isohydroxamic acid. The complex formed with iron under acidic conditions has a maximum absorption peak of 540 nm, measurable by an enzyme labeler. Therefore, 1 μmol of γ-glutamyl (ohydroxamic acid per hour per g of tissue for 1 mL of reaction system) is defined as one unit of enzyme activity (μmol h^−1^ g^−1^).

Glutamate dehydrogenase catalyzes the formation of glutamate and NAD^+^ from NH_4_^+^, α-ketoglutarate, and NADH, reducing the absorbance at 340 nm. The GDH activity was calculated by measuring the rate of decrease in absorbance at 340 nm. Therefore, consumption of 1 nmol NADH per minute per g of tissue is defined as one unit of enzyme activity (nmol min^−1^ g^−1^).

Glutamate synthase catalyzes the transfer of the amino group of glutamine to α-ketoglutarate, forming two molecules of glutamic acid; simultaneously, NADH oxidizes to form NAD^+^, and the rate of decrease in absorbance at 340 nm reflects the activity of GOGAT. Therefore, consumption of 1 nmol NADH per minute per g of tissue is defined as one unit of enzyme activity (nmol min^−1^ g^−1^).

Rubisco catalyzes the generation of 3-phosphoglyceric acid (PGA) from ribulose-1,5-bisphosphate, and PGA can produce glyceraldehyde-3-phosphate and oxidize NADH through the action of an additional 3-phosphoglycerate kinase and glyceraldehyde-3-phosphate dehydrogenase. The rate of decrease in absorbance at 340 nm reflects the activity of Rubisco. Therefore, consumption of 1 nmol NADH per minute per g of tissue is defined as one unit of enzyme activity (nmol min^−1^ g^−1^).

The reverse reaction of PPDK catalyzes the formation of pyruvate, ATP, and Pi from phosphoenolpyruvate, AMP, and PPi, and lactate dehydrogenase further catalyzes the formation of lactate and NAD^+^ from pyruvate and NADH; the rate of reduction in NADH was measured at 340 nm to calculate PPDK activity. Therefore, consumption of 1 nmol NADH per minute per g of tissue is defined as one unit of enzyme activity (nmol min^−1^ g^−1^).

Phosphoenolpyruvate carboxylase catalyzes the formation of oxaloacetate and HPO_4_^2–^ from phosphoenolpyruvate and CO_2_, and malate dehydrogenase further catalyzes the formation of malate and NAD^+^ from oxaloacetate and NADH. The rate of NADH reduction was measured at 340 nm to calculate the PEPC activity. Therefore, consumption of 1 nmol NADH per minute per g of tissue is defined as one unit of enzyme activity (nmol min^−1^ g^−1^).

### 4.4. Statistical Analyses

Data were analyzed using SPSS 22.0 (IBM, Inc., Armonk, NY, USA). To determine whether a year by treatment interaction affected the parameters of interest, a three-way mixed effects ANOVA was conducted, with year (Y), density (D), topsoil (S), and interactions (Y × D, Y × S, D × S, Y × D × S) considered as fixed effects, and replication as a random effect. In this study, the leaf activities of key N metabolism enzymes and photosynthetic enzymes were analyzed on the basis of one year of data, so the effect of the year factor was excluded. Some of the measured parameters showed a significant year by treatment interaction (Y × D, Y × S, D × S, Y × D × S) (Table S4); therefore, the analysis of all measured parameters was conducted separately for each year. One-way ANOVA was used to assess the significance of different topsoil depths on grain yield, NAR of leaf area, LAPE, Pn, and activities of key N metabolism enzymes and photosynthetic enzymes, and the least significant difference method (LSD) was used for multiple comparisons at a significance level of 0.05.

Pearson correlations were performed to assess relations between grain yield, NAR of leaf area, LAPE, Pn, and activities of key N metabolism enzymes and photosynthetic enzymes. We used structural equation models (SEM) to test the hypothetical model that the main physiological and biochemical traits would affect leaf N and C accumulation and then drive NAR of leaf area and LAPE and ultimately grain yield. The SEM was conducted with the “lavaan” package (0.6-16) in R v. 4.3.1 [79].

## 5. Conclusions

We confirmed that reducing topsoil depth significantly reduces maize grain yield while increasing the uncertainty of maize yield. When the topsoil depth was reduced from 50 cm to 10 cm it resulted in yield losses of 35.2–44.2%. The yield losses at decreasing topsoil depth were associated with impaired leaf function, decreased photosynthetic enzyme activities and photosynthetic rates, blocked N metabolism processes, and decreased C and N accumulation. Additionally, we found that yield loss and loss of leaf physiological and biochemical functions in maize in the individual planting pattern (15,000 plants ha^−1^) were almost the same as those under the population pathway (75,000 plants ha^−1^). Therefore, eliminating intraspecific competition at low crop density does not reduce yield loss due to decreased topsoil depth. We suggest that a topsoil depth of 30 cm is the threshold for the normal growth and development of individual maize. Restoration and protection of degraded soils is an important way to achieve sustainable, high agricultural yields in Northeast China.

## Figures and Tables

**Table 1:** Components of grain yield at different maize densities and topsoil depths. Values are the mean ± SD, *n* = 3. Different lowercase letters within a year for a variable indicate significant differences among treatments at *p* < 0.05, according to LSD analysis.

Year	Treatments	Double Cob Rate (%)	Kernels per Plant	1000-Kernel Weight (g)
2021	D_1_S_1_	22.2 ± 19.2 c	777.2 ± 61.8 c	368.4 ± 6.2 a
	D_1_S_2_	55.6 ± 19.3 b	962.2 ± 20.1 b	351.8 ± 4.1 a
	D_1_S_3_	100.0 ± 0 a	1171.3 ± 55.6 a	366.9 ± 11.1 a
	D_1_S_4_	100.0 ± 0 a	1238.0 ± 59.4 a	354.2 ± 5.2 a
	D_1_S_5_	100.0 ± 0 a	1248.8 ± 11.8 a	352.7 ± 7.7 a
	D_2_S_1_	0	458.3 ± 24.2 c	286.0 ± 9.5 b
	D_2_S_2_	0	514.7 ± 15.1 b	325.7 ± 3.5 a
	D_2_S_3_	0	577.7 ± 16.0 ab	348.7 ± 3.6 a
	D_2_S_4_	0	618.5 ± 14.3 a	327.1 ± 9.0 a
	D_2_S_5_	0	610.6 ± 16.2 a	337.0 ± 8.3 a
2022	D_1_S_1_	11.1 ± 38.5 c	760.0 ± 116.0 c	325.5 ± 1.8 a
	D_1_S_2_	44.4 ± 38.5 b	1008.0 ± 156.0 bc	312.3 ± 4.3 a
	D_1_S_3_	100.0 ± 0 a	1170.0 ± 66.4 ab	306.1 ± 4.2 a
	D_1_S_4_	100.0 ± 0 a	1344.3 ± 18.8 a	315.2 ± 6.3 a
	D_1_S_5_	100.0 ± 0 a	1374.7 ± 45.9 a	322.6 ± 4.3 a
	D_2_S_1_	0	438.1 ± 16.9 c	239.9 ± 3.4 c
	D_2_S_2_	0	511.2 ± 13.7 b	284.0 ± 4.3 c
	D_2_S_3_	0	609.9 ± 19.9 a	284.5 ± 6.2 bc
	D_2_S_4_	0	618.4 ± 20.8 a	287.0 ± 6.4 ab
	D_2_S_5_	0	603.3 ± 11.7 a	302.2 ± 4.8 a

**Table 2:** Treatment combinations based on two maize planting densities and five topsoil depths.

Density (Plants ha^−1^)	Topsoil Depth (cm)				
	10 (S_1_)	20 (S_2_)	30 (S_3_)	40 (S_4_)	50 (S_5_)
15,000 (D_1_)	D_1_S_1_	D_1_S_2_	D_1_S_3_	D_1_S_4_	D_1_S_5_
75,000 (D_2_)	D_2_S_1_	D_2_S_2_	D_2_S_3_	D_2_S_4_	D_2_S_5_

## Data Availability

Data is contained within the article or Supplementary Material.

## Supplementary Materials

The following supporting information can be downloaded at: https://www.mdpi.com/article/10.3390/plants14162470/s1, Figure S1: Process of building experimental plots with different topsoil depths; Table S1: Dates of samples and maize growth stages in 2021 and 2022; Table S2: Leaf area at different maize densities and topsoil depths in 2021 and 2022; Table S3: Dry matter weight at different maize densities and topsoil depths in 2021 and 2022; Table S4: Multifactor mixed effects ANOVA probability results, where year (Y), density (D), topsoil (S), and interactions (Y × D, Y × T, D × T, Y × D × T) were considered fixed effects.
