# Supplementary material for: Elimination of Intraspecific Competition Does Not Improve Maize Leaf Physiological and Biochemical Responses to Topsoil Degradation
Source: Plants (Basel). 2025 Aug 9;14(16):2470. doi: 10.3390/plants14162470 (PMC12389017; doi:10.3390/plants14162470)
Supplement: Supplementary file 1 [file plants-14-02470-s001.zip › plants-3775912-supplementary.pdf]

Appendix A. Supporting information

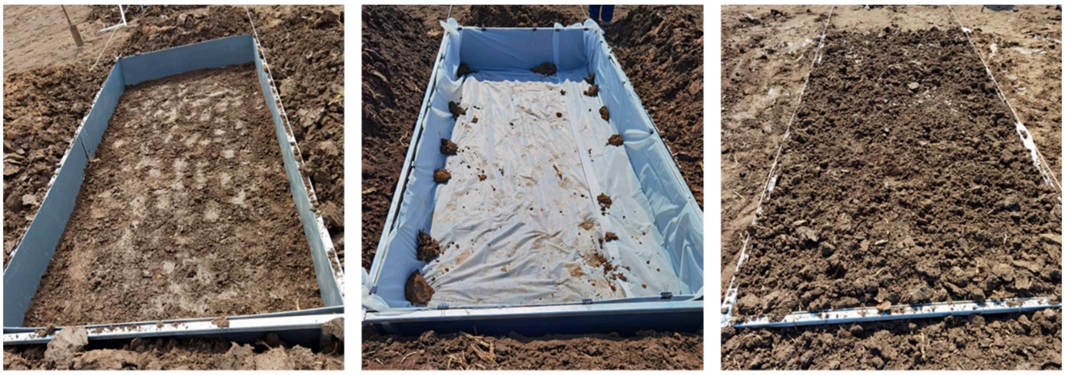

(A) (B) (C)

Figure S1. Process of building experimental plots with different topsoil depths.

(A) Topsoil is removed to the required depth, and the flat bottom surface is compacted.

(B) Plot bottom is covered with nylon mesh (0.074 mm).

(C) Area above the nylon mesh is filled with the removed soil, watered, and allowed to settle.

Table S1. Dates of samples and maize growth stages in 2021 and 2022. ES, emergence of seedlings; VT, tassel stage; R6, maturity stage.

Year	Date		
	ES	VT	R6
2021	May.25	Aug.1	Oct.2
2022	May.20	Aug.5	Sept.30

Table S2 Leaf area at different maize densities and topsoil depths in 2021 and 2022. Density: D_1, 15,000 plants ha^{-1}; D_2, 75,000 plants ha^{-1}. Topsoil depth: S_1, 10 cm; S_2, 20 cm; S_3, 30 cm; S_4, 40 cm; and S_5, 50 cm. Maize growth stage: VT, tassel stage; R6, maturity stage.

**Year**	**Density**	**Topsoil depth**	**Leaf area (cm^2 plant^{-1})**	
			**VT**	**R6**
2021	D_1	S_1	8849.6	3830.0
		S_2	9331.2	3913.0
		S_3	9566.0	4605.2
		S_4	9948.5	4540.9
		S_5	10042.1	4867.8
	D_2	S_1	6234.2	2724.8
		S_2	6930.0	3238.2
		S_3	7312.4	3646.3
		S_4	7689.9	3699.4
		S_5	7725.9	3827.2
2022	D_1	S_1	7693.3	2582.8
		S_2	8277.3	3741.7
		S_3	9156.9	3595.6
		S_4	9779.2	3692.5
		S_5	9799.9	3899.9
	D_2	S_1	6056.4	2611.2
		S_2	6650.7	2426.3
		S_3	7231.8	2979.2
		S_4	7812.4	2980.9
		S_5	7957.3	3144.3

Table S3. Dry matter weight at different maize densities and topsoil depths in 2021 and 2022. Density: D_1, 15,000 plants ha^{-1}; D_2, 75,000 plants ha^{-1}. Topsoil depth: S_1, 10 cm; S_2, 20 cm; S_3, 30 cm; S_4, 40 cm; and S_5, 50 cm. Maize growth stage: VT, tassel stage; R6, maturity stage.

**Year**	**Density**	**Topsoil depth**	**Dry matter weight (g plant^{-1})**	
			**VT**	**R6**
2021	D_1	S_1	170.1	490.9
		S_2	193.5	558.4
		S_3	252.4	671.4
		S_4	256.3	694.6
		S_5	266.7	698.2
	D_2	S_1	90.3	234.9
		S_2	111.6	290.4
		S_3	133.2	331.5
		S_4	164.5	332.1
		S_5	167.2	339.5
2022	D_1	S_1	187.8	569.5
		S_2	213.0	706.5
		S_3	253.6	743.2
		S_4	267.3	742.9
		S_5	259.3	773.2
	D_2	S_1	88.0	173.0
		S_2	99.9	205.6
		S_3	117.7	261.5
		S_4	144.2	271.7
		S_5	150.9	295.1

Table S4. Multifactor mixed effects ANOVA probability results, where year (Y), density (D), topsoil (S), and interactions (Y×D, Y×T, D×T, Y×D×T) were considered fixed effects. The activities of key enzymes of nitrogen metabolism and photosynthesis were determined in only one year of the study, so the effect of year factor was excluded. NR, nitrate reductase (nmol min^{-1} g^{-1}); GS, glutamine synthetase (μmol h^{-1} g^{-1}); GDH, glutamate dehydrogenase (nmol min^{-1} g^{-1}); GOGAT, glutamate synthase (nmol min^{-1} g^{-1}); Rubisco, ribulose−1,5−bisphosphate carboxylase (nmol min^{-1} g^{-1}); PPDK, pyruvate orthophosphate dikinas (nmol min^{-1} g^{-1}); PEPC, phosphoenolpyruvate carboxylase (nmol min^{-1} g^{-1}). *$P < 0.05$; **$P < 0.01$; ***$P < 0.001$; ns, no significant effect.

**Parameters**	***F*-values**						
	**Years (Y)**	**Density (D)**	**Topsoil (S)**	**Y×D**	**Y×S**	**D×S**	**Y×D×S**
Grain yield (g $plant^{-1}$)	10.3**	594.9***	32.3***	0.1ns	0.6ns	5.4**	0.5ns
Net assimilation rate of leaf area (g m^{-2} d^{-1})	299.8***	4346.0***	337.2***	260.8***	67.9***	259.4***	51.5***
Leaf area production efficiency (kg m^{-2})	3.3ns	161.1***	15.7***	11.5**	0.5ns	0.8ns	0.6ns
N							
Leaf nitrogen accumulation (g $plant^{-1}$)	305.5***	863.9***	101.1***	1.8ns	1.0ns	3.8*	3.8*
Leaf carbon accumulation (g $plant^{-1}$)	143.0***	623.1***	85.9***	77.6***	1.5ns	2.7*	2.2ns
Photosynthesis rate (mean) (μmol CO_2 m^{-2} s^{-1})	2.3ns	827.2***	177.7***	20.1***	2.1ns	2.0ns	2.5ns
NR activities (nmol min^{-1} g^{-1} NADH)		43.9***	36.7***			1.8ns	
GS activities (μmol h^{-1} g^{-1} γ-GIA)		7.0*	14.2***			0.2ns	
GDH activities (nmol min^{-1} g^{-1} NADH)		99.0***	77.2***			3.4*	

GOGAT activities (nmol min^{-1} g^{-1} NADH)	43.6***	76.3***		7.1**
Rubisco (nmol min^{-1} g^{-1})	69.7***	37.4***		4.8**
PPDK (nmol min^{-1} g^{-1})	203.3***	65.3***		15.0***
PEPC (nmol min^{-1} g^{-1})	518.6***	115.7***		4.6**
